# Impulsivity, time perception and non-suicidal self-injury in adolescents: from behavioral and fNIRS evidence

**DOI:** 10.3389/fpsyt.2026.1773287

**Published:** 2026-04-22

**Authors:** He He, Lipeng Chen, Yuxuan Wu, Linling Hu, Lan Hong, Ke Zhao, Dongwu Xu

**Affiliations:** 1 School of Mental Health, Wenzhou Medical University, Wenzhou, China; 2 School of Psychology, Shanghai Normal University, Shanghai, China; 3 Department of Clinical Psychology, Lishui Second People’s Hospital Affiliated to Wenzhou Medical University, Lishui, China

**Keywords:** adolescents, fNIRS, impulsivity, non-suicidal self-injury, time perception

## Abstract

**Background:**

Elevated impulsivity and temporal processing deficits are key risk factors for adolescent non-suicidal self-injury (NSSI); however, their joint neural substrates and specific contributions to NSSI remain inadequately characterized. This study aimed to elucidate the behavioral and neural interplay between these cognitive domains in adolescents with NSSI.

**Methods:**

We recruited 44 adolescents with NSSI and 37 typically developing (TD) controls. Participants completed the Barratt Impulsiveness Scale (BIS-11), a Choice Delay Task (CDT), and time perception assessments (discrimination and estimation tasks). A subsample (33 NSSI, 30 TD) subsequently underwent functional near-infrared spectroscopy (fNIRS) to monitor prefrontal hemodynamic responses during an oddball task, a paradigm assessing inhibitory control.

**Results:**

Compared to TD controls, adolescents with NSSI exhibited significantly elevated trait impulsivity (BIS-11), heightened delay aversion (preference for immediate rewards in CDT), impaired short-interval temporal discrimination (600ms), and a consistent underestimation of time intervals (7s, 12s, 34s, and 90s). Stepwise regression analysis identified BIS-11 scores, 600ms discrimination thresholds, and 90s estimation bias as significant predictors of NSSI. Neuroimaging revealed that the NSSI group showed lower accuracy on the oddball task and significant hypoactivation in the left dorsolateral prefrontal cortex (L-DLPFC). Crucially, reduced L-DLPFC activation was associated with greater time estimation errors (at 12s and 34s) and increased NSSI.

**Conclusion:**

Adolescents with NSSI display a distinct neurocognitive phenotype characterized by high impulsivity and distorted time perception. These deficits may be associated with reduced activation in the L-DLPFC. Our findings suggest that NSSI involves a dual failure of inhibitory control and temporal processing, indicating that interventions targeting prefrontal regulation may offer novel strategies for risk reduction.

## 1 Introduction

Non-suicidal self-injury (NSSI), the deliberate destruction of one’s own body tissue without suicidal intent (1), poses a serious threat to the physical and mental health of adolescents. In China, the estimated lifetime prevalence among youth is an alarming 24.7% and trending upwards (2). NSSI is strongly associated with other mental health disorders, such as depression and anxiety (3, 4), and is a potent risk factor for future suicidal ideation and behavior (5). Given its severe impact, elucidating the neuropsychological mechanisms underlying NSSI is a critical public health priority.

Impulsivity is a core vulnerability factor for NSSI (6). It is characterized by rapid, unplanned reactions to internal or external stimuli without regard for negative consequences (7). Adolescents with high impulsivity are significantly more likely to engage in NSSI (8). Neuroimaging studies have linked this behavioral disinhibition to abnormal activation in the prefrontal cortex (PFC), a region essential for executive control and decision-making (9). Trait impulsivity, as assessed by self-report measures such as the BIS-11, reflects a stable disposition toward unplanned, rapid behavior and sensation seeking. Inhibitory control, by contrast, refers to the specific executive capacity to suppress prepotent or contextually inappropriate responses. It comprises two subdomains: reactive inhibition—the ability to stop an ongoing response when a stop signal appears—and proactive inhibition—the ability to prepare for stopping in advance based on contextual cues (10, 11). The oddball task used in the present study primarily measures reactive inhibition, as participants must suppress the prepotent response to frequent stimuli when a rare deviant appears (12). Although these represent distinct neurocognitive constructs (13, 14), both have been implicated in NSSI. Specifically, for individuals experiencing overwhelming negative affect, NSSI may serve as a rapid means to achieve relief—functioning, in a sense, as a salient reward. In this framework, high trait impulsivity provides the motivational drive to obtain the reward, predisposing individuals to seek immediate relief as a first-line response to distress. Concurrently, poor inhibitory control fails to intercept or override this prepotent impulse to self-harm, thereby allowing the behavior to be enacted. Theoretical models, such as the Dual Systems Model, posit that adolescent risk behaviors result from heightened impulsive reactivity(hyperactive socio-emotional system)coupled with immature or impaired inhibitory control (hypoactive cognitive control system) (15). Empirical studies support this in NSSI, showing that patients often exhibit both high trait urgency and deficits in executive inhibition tasks (16).

In addition, another factor that may also participate in this process is time perception—the subjective experience of the passage of time (17). The “internal clock” model suggests that distorted time perception is fundamental for cognitive functions like decision-making and future planning (18, 19). Previous studies have found that individuals with NSSI exhibit underestimation of time intervals, which may be associated with dysregulation of the reward system (20). However, based on the internal clock model, impulsivity-related mechanisms would also predict overestimation (21, 22), creating theoretical ambiguity that needs empirical examination of the interplay between time perception deficits and other cognitive functions in NSSI.

Furthermore, both impulsivity and time perception rely on overlapping neural circuits, particularly the fronto-striatal loops involving the dorsolateral prefrontal cortex (DLPFC) (23, 24). While previous fNIRS studies have identified reduced DLPFC activation in NSSI during cognitive tasks (25, 26), none have simultaneously examined how these neural deficits relate to temporal processing impairments.

Therefore, the present study aims to explore the cognitive and neural mechanisms of NSSI by integrating behavioral assessments with functional near-infrared spectroscopy (fNIRS). We hypothesize that (1): Adolescents with NSSI will exhibit elevated impulsivity and distorted time perception compared to controls; and (2) These deficits will be underpinned by hypoactivation in the prefrontal cortex, specifically the L-DLPFC.

## 2 Methods

### 2.1 Participants

A total of 85 adolescents were recruited from psychiatric wards, outpatient clinics, and the local community between July 2023 and January 2024. Four participants from the NSSI group were excluded from the final analysis as they withdrew from the study after completing the questionnaire assessments but before participating in the behavioral paradigms. The final sample included 81participants: 44 adolescents with NSSI and 37 typically developing (TD) controls.

Inclusion criteria for the NSSI group were (1): age 13–18 years (2); right-handed (3); meets DSM-5 diagnostic criteria for NSSI-D (≥5 episodes in the past 12 months) with symptom stability confirmed by at least two psychiatrists (4); absence of suicidal intent during NSSI. Exclusion criteria for the NSSI group were (1):history of severe physical diseases, brain trauma, epilepsy, or other known severe neurological diseases or brain organic diseases (2); history of severe mental disorders such as schizophrenia, psychotic disorders, or intellectual disability (3); inability to cooperate with experimental procedures, or withdrawal from the study. The NSSI participants had a comorbid mood disorder: 35 with major depressive disorder and 9 with bipolar disorder (current depressive episode). All were receiving pharmacological treatment with SSRIs and/or mood stabilizers at the time of assessment (see **Table 1**).

**Table 1:** Medication regimens of NSSI participants by diagnosis.

Diagnosis	Medication regimen	N	% (Within diagnosis)
MDD (N = 35)	SSRI	12	34.3
	SSRI + mood stabilizer	23	65.7
BD (N = 9)	mood stabilizer	2	22.2
	SSRI + mood stabilizer	7	77.8

SSRIs included fluoxetine, sertraline, escitalopram, paroxetine. Mood stabilizers included lamotrigine, valproate, lithium.

The typically developing (TD) controls were recruited from local middle schools through online and offline advertisements. Inclusion criteria for the TD group were (1): age 13–18 years (2); right-handed (3); no history of NSSI or any psychiatric disorder (4); no history of psychiatric treatment. Exclusion criteria for the TD group were (1): any current or past psychiatric diagnosis (2); history of organic brain diseases (3); inability to cooperate with experimental procedures.

It should be noted that a subset of these participants (33 NSSI, 30 TD) agreed to undergo the fNIRS neuroimaging protocol. There were no significant demographic differences between the total sample and the fNIRS subsample.

The study was approved by the Clinical Research Committee of the First Affiliated Hospital of Wenzhou Medical University (KY2023-039). All participants and their legal guardians provided written informed consent.

### 2.2 Psychological assessments

#### 2.2.1 Clinical assessment of NSSI

The Adolescent Non-Suicidal Self-Injury Assessment Questionnaire was used to evaluate the frequency, severity, and functions of NSSI behaviors (Cronbach’s alpha =0.913). The questionnaire comprises behavior and function subscales, both are scored on a scale of 0-4 (27). For this study, two additional items were included to specifically assess (1): the time interval between the urge to self-injure and the enactment of the behavior, and (2) the subjective level of pain perception during NSSI.

#### 2.2.2 Impulsivity assessment

Trait impulsivity was assessed using the Barratt Impulsiveness Scale (BIS-11) (28) (Cronbach’s alpha =0.948). This 30-item self-report measure evaluates three sub-dimensions of impulsivity: Cognitive, Motor, and Non-planning. Higher total scores indicate higher levels of trait impulsivity.

#### 2.2.3 Mental health and emotion regulation

Depressive symptoms were assessed using the Beck Depression Inventory (BDI) (29) (Cronbach’s alpha =0.967). Anxiety symptoms were evaluated using the Generalized Anxiety Disorder-7 (GAD-7) (30) (Cronbach’s alpha =0.954). General psychological distress was measured using the Psychological Distress Scale (31) (Cronbach’s alpha =0.977). Emotion regulation strategies were assessed using the Emotion Regulation Questionnaire for Children and Adolescents (ERQ-CA) (32) (Cronbach’s alpha =0.707), focusing on cognitive reappraisal and expressive suppression.

### 2.3 Behavioral paradigms

#### 2.3.1 Choice delay task

To measure delay aversion, participants made 10 choices between a smaller, immediate reward (1 point after a 2-s delay) and a larger, delayed reward (2 points after a 30-s delay) (33). The number of selections for the larger, delayed reward served as the primary outcome measure, with fewer selections indicating higher impulsivity. All behavioral tasks were programmed and presented using PsychoPy (Version 2022.2.1).

#### 2.3.2 Time discrimination task

Participants were presented with two sequential circular stimuli of differing durations and asked to identify the longer one. The task used a staircase procedure, adjusting the comparison duration by 20ms based on response accuracy. Two blocks were completed with standard durations of 1400ms and 600ms, respectively (34).

#### 2.3.3 Time estimation task

Participants were shown a circle for four different durations (7s, 12s, 34s, and 90s), with each duration presented three times in random order. After each presentation, they were asked to numerically estimate the duration in seconds. The deviation from actual time served as the measure of accuracy (34).

### 2.4 fNIRS data acquisition and analysis

**Figure 1:**
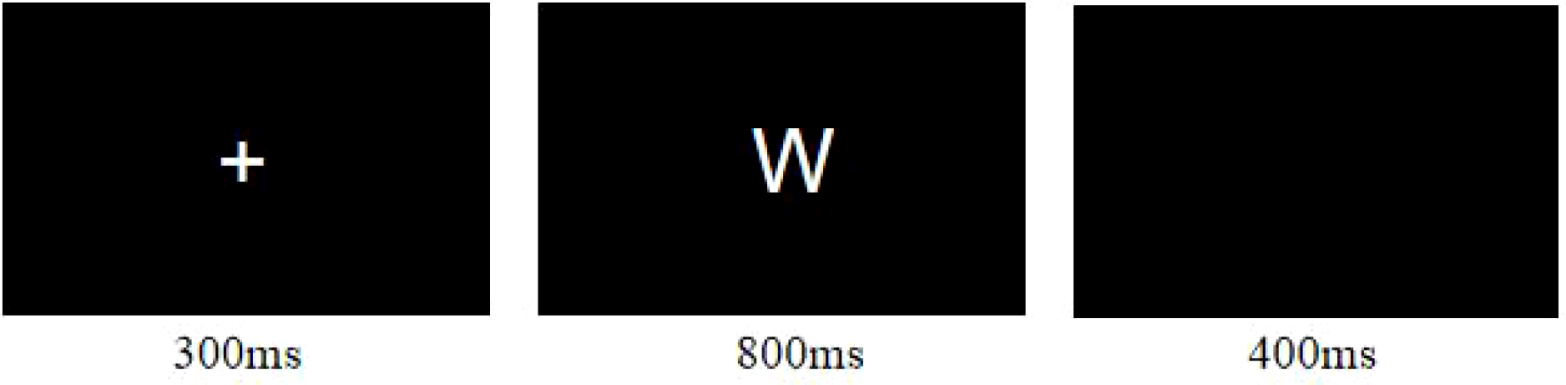
The oddball task. The picture demonstrate a single trial of the Oddball task which contains 4 blocks, and every block contain 60 trials, participants will rest for 10 seconds between every 2 blocks.

Participants performed a two-choice Oddball task to assess inhibition control during fNIRS recording. The task included frequent standard stimuli (letter “W”, 83%, 50 trials per block) and rare deviant stimuli (letter “M”, 17%, 10 trials per block). Stimuli were presented in a pseudo-random order for a duration of 800ms. Participants were instructed to press “F” for standard stimuli and “J” for deviant stimuli as quickly and accurately as possible. The session comprised 4 blocks of 60 trials each. For details, see **Figure 1**.

**Figure 2:**
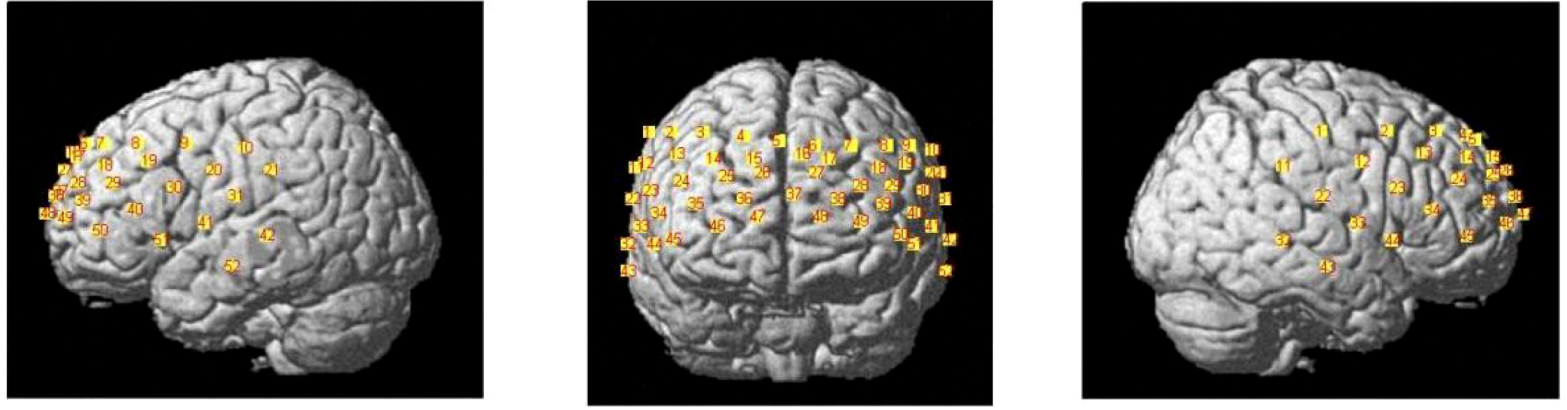
The fNIRS cortical localization map.

The fNIRS data were recorded using a Hitachi ETG-4100 optical topography system with a 52-channel array (3×11 probes). The positions of the 52 channels were confirmed using a 3D digitizer to register the locations of the optode probe and scalp landmarks (nasion, central midline point, and left/right mastoids) for cortical region localization (as shown in **Figure 2**). After registering the optode positions from the International 10–20 system to the Montreal Neurological Institute (MNI) space, each measurement channel was assigned corresponding MNI coordinates, thereby enabling the inference of the specific cortical regions it covered (35). The sampling rate was 10 Hz.

The Hitachi ETG-4100 system’s built-in monitoring software generates real-time NIRS time-series traces, which were used to assess signal quality for each channel. Channels with poor signal quality—due to factors such as poor optode-scalp contact, signal saturation, high-frequency noise, or obvious motion artifacts—were automatically flagged by the system. Through visual inspection of these flagged channels, we confirmed their inadequacy and subsequently excluded them from further analysis. Channels 9, 10, 11, 20, 21, 31, 32, and 42 were excluded and all remaining 44 channels were retained for subsequent statistical analysis (36).

The fNIRS data were analyzed using NIRS-SPM (Statistical Parametric Mapping for Near-infrared Spectroscopy) software implemented in MATLAB 2013a. The preprocessing pipeline included (1): Hemodynamic Response Function (HRF) smoothing to remove high-frequency noise; and (2) Wavelet-MDL detrending to eliminate physiological artifacts and machine drift. Oxygenated hemoglobin (HbO) concentration changes were selected as the primary indicator of neural activation due to its high sensitivity to cerebral blood flow changes. A General Linear Model (GLM) was applied to estimate cortical activation. A task-related regressor modeling the hemodynamic response during the Oddball task blocks was convolved with the canonical hemodynamic response function. A 30-second resting-state period immediately preceding the first task block was modeled as the baseline reference. The contrast of interest was [Oddball Task Blocks vs Baseline]. Beta values (*β*) for this contrast were extracted for each channel, representing task-related HbO concentration changes relative to the pre-task resting baseline.

### 2.5 Statistical analysis

Statistical analyses were performed using IBM SPSS Statistics 26.0 (IBM Corp., Armonk, NY, USA). The significance level was established at α<0.05(two-tailed) for all tests. Prior to analysis, all continuous variables were screened for outliers (defined as values exceeding ±3 standard deviations from the mean) and assessed for normality using the Shapiro-Wilk test. Levene’s test was utilized to verify the assumption of homogeneity of variance. For data adhering to normal distribution assumptions, parametric tests were employed; otherwise, non-parametric equivalents or logarithmic transformations were applied. Descriptive statistics are presented as mean ± standard deviation (*M ± SD*) for continuous variables and frequency for categorical (percentage) variables.

#### 2.5.1 Demographic and psychological measures

Group differences in demographic characteristics and psychological scale scores between the NSSI and Control (TD) groups were examined using independent samples *t*-tests for continuous variables and Chi-square (*χ2*) tests for categorical variables.

#### 2.5.2 Analysis of behavioral paradigms

To isolate the effects of NSSI behavior from potential developmental confounds, Age was included as a covariate in all behavioral analyses. For the choice delay task, a one-way ANCOVA was conducted with group as the between-subjects factor. The frequency of choosing the immediate reward served as the dependent variable. A mixed-design ANCOVA was performed for two time tasks. The between-subjects factor was Group (NSSI vs. Control), and the within-subjects factor was Condition (1400ms/600ms for discrimination) or Duration (7s/12s/34s/90s for estimation).

For repeated measures, Mauchly’s test of sphericity was conducted. If the assumption of sphericity was violated, degrees of freedom were adjusted using the Greenhouse-Geisser correction. Significant main effects and interactions were followed up with Bonferroni-corrected *post-hoc* comparisons. Effect sizes for ANOVA/ANCOVA were reported as partial eta squared(*η^2^*).

#### 2.5.3 fNIRS data analysis

For the fNIRS data, beta values (*β*) representing oxygenated hemoglobin (HbO) concentration changes were extracted for each channel. Independent samples t-tests were used to compare activation differences between groups for each channel. To address the multiple comparison problem inherent in multi-channel fNIRS data, we focused on regions of interest (ROIs) identified based on previous literature (specifically the DLPFC) and applied the False Discovery Rate (FDR) correction (Benjamini-Hochberg procedure) where applicable for channel-wise analyses. According to previous literature, Dahlgren and colleagues reported altered prefrontal cortex activation during cognitive interference in individuals with NSSI (24), while another study demonstrated a link between DLPFC activity and risk for suicidal ideation and self-injury (25). Furthermore, the DLPFC is a core node in fronto-striatal circuits underlying both inhibitory control (17) and time perception (23), making it a theoretically justified ROI for examining the interplay between these cognitive domains in NSSI.

#### 2.5.4 Correlation and regression analyses

To explore the brain-behavior-symptom mechanism, two separate partial correlation analyses were conducted due to different sample sizes, controlling for age and depression (BDI scores). To identify predictors of NSSI, a stepwise multiple linear regression analysis (bidirectional) was performed. Variables demonstrating significant correlations with NSSI (based on the full sample of 81) were entered as independent predictors(Include BIS-11, 600ms Threshold, 7s Time Estimation, 90s Time Estimation, and CDT delayed choice frequency), and the criteria for variable entry was p<0.05 and removal criterion was p>0.1. Collinearity diagnostics were systematically checked using the Variance Inflation Factor (VIF), with VIF <5 considered acceptable to rule out multicollinearity issues.

## 3 Result

### 3.1 Demographic and clinical characteristics

The final sample for the behavioral study comprised 81 adolescents: 44 with NSSI (86.4% female, *M_ag_*_e_ = 15.02 ± 1.36) and 37 typically developing (TD) controls (86.5% female, *M_age_* = 16.43 ± 0.05). Demographic and clinical data are summarized in **Table 2**. However, the Control group was slightly older than the NSSI group (t=-5.99, p<0.001); thus, age was included as a covariate in all subsequent analyses.

**Table 2:** Demographic characteristics, clinical scores, and behavioral performance of NSSI and TD.

Variable	NSSI (N = 44)	TD (N = 37)	t/χ^2^/F	d/η^2^
Gender(Female/Male)	38/6	32/5	0.01	-
Age(years)	15.02 ± 1.36	16.43 ± 0.05	-5.99***	-
(GAD-7)Anxiety	13.52 ± 5.55	3.59 ± 3.39	9.03***	2.14
(BDI)Depression	32.41 ± 10.61	6.27 ± 7.86	12.90***	2.87
Psychological distress	50.16 ± 9.27	25.32 ± 11.50	9.70***	2.37
BIS				
Cognitive Impulsiveness(CI)	33.02 ± 6.54	25.19 ± 6.59	4.78***	1.19
Motor Impulsiveness (MI)	31.91 ± 6.64	25.51 ± 8.72	3.38***	0.82
Noplanning Impulsiveness (NI)	37.59 ± 6.30	27.29 ± 8.40	6.19***	1.38
Total scores	102.52 ± 14.87	78.00 ± 19.50	6.04***	1.34
Emotion Regulation				
Cognitive reappraisal	16.25 ± 4.82	20.95 ± 4.47	-3.96***	1.01
Expression inhibition	13.70 ± 2.81	12.65 ± 2.69	1.71	0.38
Choice delay				
Delayed Choice Frequency	5.20 ± 3.81	7.95 ± 2.81	14.59***	0.16

Data are presented as Mean ± SD. F statistics for CDT are derived from ANCOVA controlling for age. **p < 0.001.

Clinically, the NSSI group exhibited significantly higher scores on measures of anxiety (GAD-7), depression (BDI), and psychological distress compared to the Control group (p<0.001 for all). Regarding emotion regulation, the NSSI group scored significantly lower on Cognitive Reappraisal (t=−3.96, p<0.001) but did not differ significantly on Expressive Suppression (t=1.71, p=0.09).

### 3.2 Behavioral results

The NSSI group demonstrated elevated impulsivity (self-report and behavioral results) across both subjective and objective measures. On the self-report BIS-11, the NSSI group scored significantly higher than controls on the total score (t=6.04, p<0.001, d=1.43) as well as on the AI, MI, and NI subscales (see **Table 2**).

In the choice delay task (CDT), after controlling for age, the NSSI group selected the delayed reward significantly less frequently than the TD group (5.20 ± 3.81 vs. 7.95 ± 2.81; F (1,78)=14.59, p<0.001, η^2^ = 0.16). This reduced capacity to delay gratification indicates a heightened delay aversion and elevated behavioral impulsivity in adolescents with NSSI.

**Figure 3:**
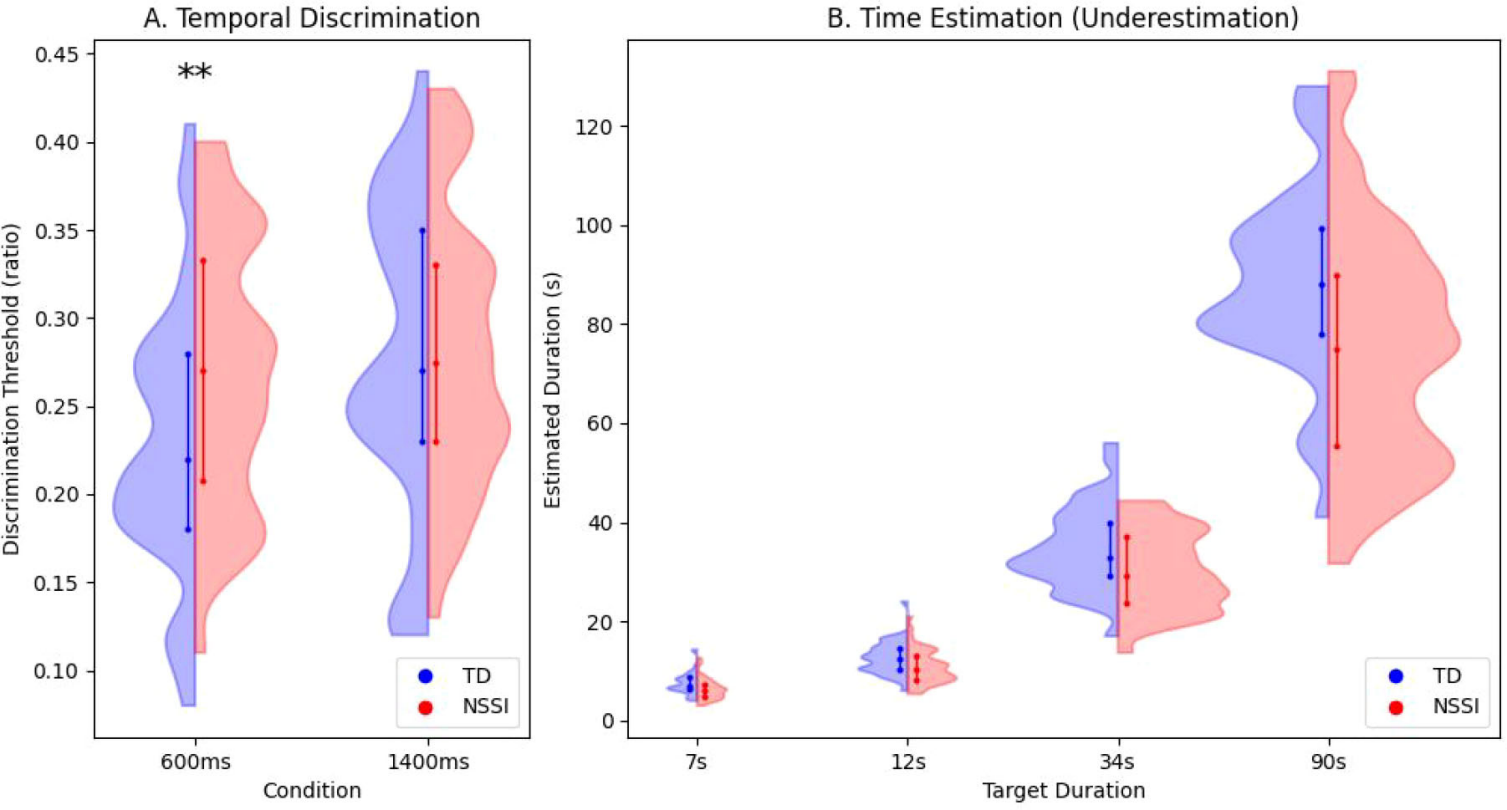
Time tasks behavioral results. Performance on time perception tasks. **(A)** Temporal discrimination thresholds. The NSSI group exhibited a significantly higher discrimination threshold (poorer sensitivity) specifically in the 600ms condition (p<0.01). **(B)** Time estimation task. The NSSI group consistently underestimated durations across all intervals (7s, 12s, 34s, 90s) compared to controls. The dashed lines represent the objective target durations. Error bars represent Standard Error (SE). *p < 0.05, **p < 0.01.

In the time discrimination task, a repeated measures ANOVA showed a significant main effect of Group (F (1,78) = 8.22, p < 0.01, η^2^ = 0.09). Post-hoc analysis indicated a specific deficit at the short interval: the NSSI group had a significantly higher discrimination threshold in the 600ms condition (p=0.01, η^2^ = 0.08), whereas no significant difference was found in the 1400ms condition (p>0.05) (see **Figure 3**). Specifically, the NSSI group had a significantly higher discrimination threshold in the 600ms condition (t=2.40, p<0.01, d=0.54), indicating impaired temporal resolution for short intervals.

In the time estimation task, there was a significant main effect of Group (F (1,78) = 4.36, p < 0.05, η^2^ = 0.05) and a significant Group × Duration interaction (F (3,234) = 5.10, p < 0.05, η^2^ = 0.06). As illustrated in **Figure 3**, the NSSI group consistently underestimated time intervals compared to controls. Simple effects analysis showed significant underestimation by the NSSI group at all durations: 7s (p=0.03, d=0.49), 12s (p=0.02, d=0.51), 34s (p=0.02, d=0.52), and 90s (p=0.007, d=0.62). The magnitude of the deviation (underestimation) progressively increased with longer durations.

### 3.3 fNIRS results

In the fNIRS subsample (N = 63), the NSSI group showed significantly lower accuracy on the Oddball task compared to controls (0.91 ± 0.09 vs. 0.95 ± 0.03;t=2.56,p=0.013,d=0.58), reflecting impaired response inhibition. Reaction times did not differ significantly between groups (p>0.05).

**Figure 4:**
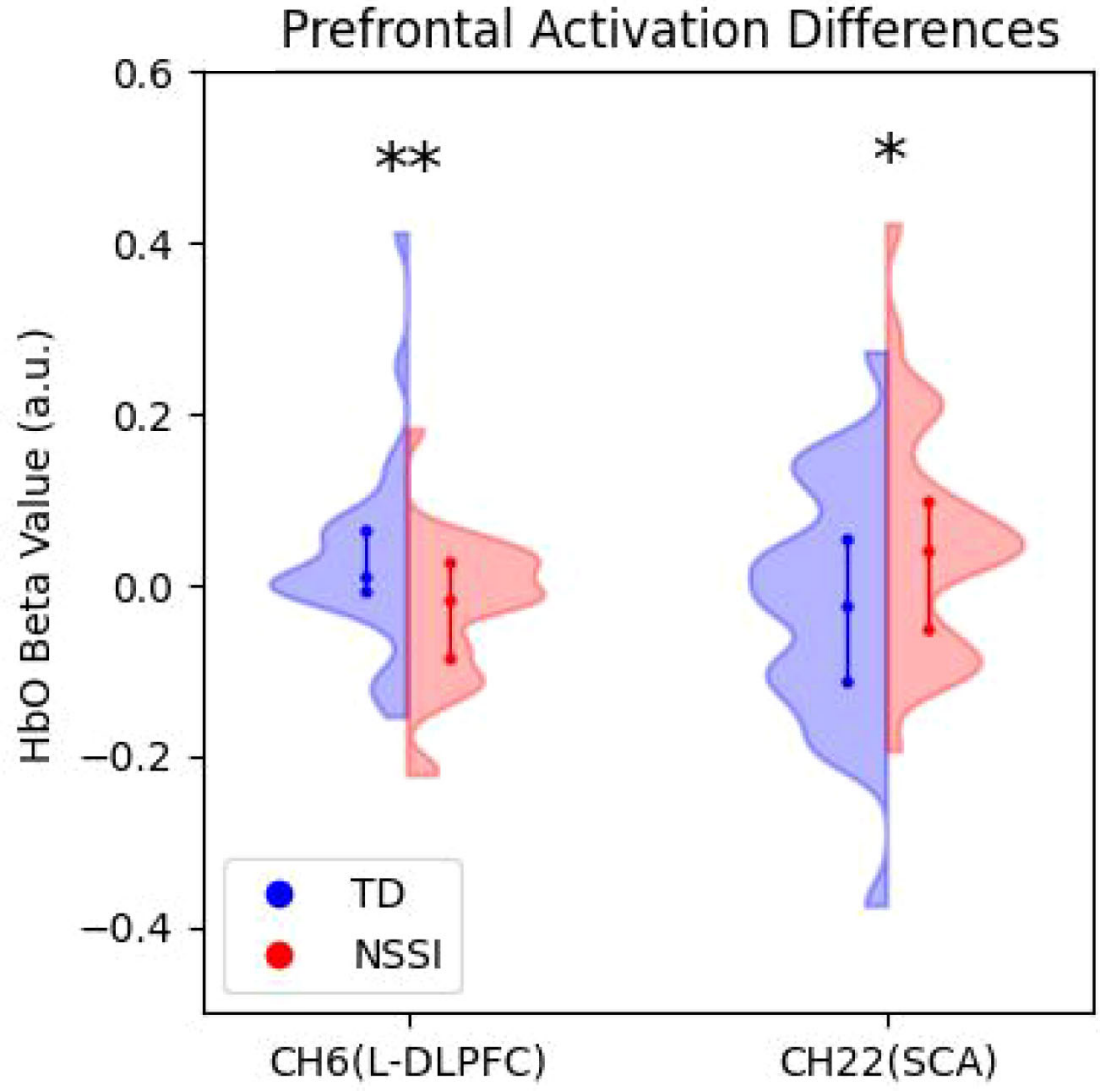
fNIRS Results. Group differences in prefrontal hemodynamic activation. Bar charts illustrate the mean oxygenated hemoglobin (HbO) beta values for regions showing significant group differences. (Left) In Channel 6 (L-DLPFC), the NSSI group showed significantly reduced activation (hypoactivation) compared to controls. (Right) In Channel 22 (Subcentral Area, SCA), the NSSI group showed significantly increased activation (hyperactivation). Error bars represent Standard Error (SE). *p < 0.05.

fNIRS analysis identified distinct patterns of hemodynamic activation (HbO β). Independent samples t-tests revealed that the NSSI group showed significantly reduced activation in Channel 6 (MNI coordinates: -12, 54, 44), corresponding to the Left Dorsolateral Prefrontal Cortex(L-DLPFC) compared to controls (t=−2.50, p=0.015, d=0.63). Furthermore, the NSSI group exhibited significantly increased activation in Channel 22 (MNI coordinates: 70, -18, 21), located in the Subcentral Area (SCA) (t=−2.12, p=0.038, d=0.54). No other channels survived significance testing(see **Figure 4**).

### 3.4 Correlations between behavior, symptoms, and brain

Partial correlation analysis (controlling for age) revealed the relationships among various variables (see **Table 3**). NSSI was positively correlated with BIS-11 scores (r=0.37, p<0.01) and the 600ms discrimination threshold (r=0.30, p<0.05), and negatively correlated with L-DLPFC activation (r=−0.35, p<0.05). L-DLPFC Activation (CH6) was significantly positively correlated with time estimation accuracy at 12s (r=0.35, p<0.05) and 34s (r=0.38, p<0.01). Specifically, lower L-DLPFC activation was associated with greater underestimation of time and more severe NSSI behavior. The activation of the SCA (CH22) did not show significant correlations with behavioral impulsivity or time perception measures (p>0.05).

**Table 3:** Partial correlations between NSSI, behavioral metrics, and brain activation.

Variable	1	2	3	4	5	6	7	8	9	10
1. NSSI	–									
2. BIS-11 Total	0.37**	–								
3. CDT	-0.25*	-0.31**	–							
4. 600ms Threshold	0.30*	0.34**	-0.23	–						
5. 7s Estimation	-0.24*	-0.25*	-0.02	-0.06	–					
6. 12s Estimation	-0.18	-0.24*	-0.11	-0.03	0.09**	–				
7. 34s Estimation	-0.19	-0.22	0.01	0.03	0.83**	0.86**	–			
8. 90s Estimation	-0.27*	-0.23	-0.08	-0.02	0.81**	0.86**	0.89**	–		
9. L-DLPFC (CH6)	-0.35*	-0.03	0.24	0.09	0.30	0.35*	0.38**	0.28	–	
10. SCA (CH22)	-0.04	0.26	-0.12	-0.01	-0.24	-0.13	-0.13	-0.07	0.28	–

Partial correlations between brain activation (CH6, CH22) and other variables are based on a sub-sample of 63 participants. All other correlations are based on the full sample of 81 participants. *p < 0.05, **p < 0.01.

A stepwise linear regression was conducted to determine the strongest predictors of NSSI. The final model (F(3,77)=12.61, p<0.001, R^2^ = 0.36) retained three predictors. Specifically, BIS-11 total scores (β=0.35, t=3.10, p=0.003),600ms discrimination threshold (β=0.24,t=2.21, p=0.03), 90s time estimation (β=−0.23,t=−2.19, p=0.031) of model, that all predict higher NSSI. Collinearity diagnostics indicated acceptable VIF values (all VIF <1.4) (see **Table 4**).

**Table 4:** Stepwise linear regression model predicting NSSI behavior.

Predictor	*β*	*t*	*P-value*	*r²*(change)	*F* change	VIF
Included Variables	–	–	–	–		
Impulsivity (BIS-11)	0.35	3.10	< 0.01	0.27	25.88**	1.343
600ms Threshold	0.24	2.21	< 0.05	0.04	4.22*	1.220
90s Time Estimation	-0.23	-2.19	< 0.05	0.05	4.80*	1.113
Excluded Variables	–	–	–	–		
7s Time Estimation	0.02	0.10	> 0.05	–	–	3.129
Delayed Choice Frequency	-0.18	-1.66	> 0.05	–	–	1.238

The final model summary: Total R^2^ = 0.36, F (3,77)=12.61, p<0.001. β, Standardized Coefficient. VIF, Variance Inflation Factor. Significance levels: *p < 0.05, **p < 0.01.

## 4 Discussion

The present study represents the first integrated investigation combining clinical psychological scales, behavioral paradigms and brain imaging technique to elucidate the interplay between impulsivity, time perception, and neural function in adolescents with NSSI. Our findings reveal a distinct neurocognitive phenotype in this population (1): Elevated Impulsivity: Adolescents with NSSI exhibit higher trait impulsivity and a preference for immediate rewards (2); Distorted Time Perception: They display impaired temporal discrimination for short intervals and a consistent underestimation of longer durations; and (3) Distinct Neural Signatures: These behavioral deficits were accompanied by hypoactivation of the Left Dorsolateral Prefrontal Cortex (L-DLPFC) and hyperactivation of the Subcentral Area (SCA). The result of stepwise regression analysis identified trait impulsivity as the strongest predictor of NSSI severity. Notably, temporal processing measures (specifically short-interval discrimination and long-interval underestimation) contributed additional unique variance, suggesting that time perception deficits represent a distinct cognitive mechanism independent of impulsivity. Based on these findings, we consider that the magnitude of time estimation errors, along with trait impulsivity, predicted NSSI severity, and these behavioral deficits were associated with L-DLPFC hypoactivation, supporting a synergistic model in which cognitive control failures and altered sensory processing characterize NSSI behaviors.

Consistent with the biosocial model (37) and previous meta-analyses (38), our NSSI cohort scored significantly higher on all dimensions of the BIS-11 and exhibited steeper delay aversion in the Choice Delay Task (CDT). This aligns with the conceptualization of NSSI as a maladaptive strategy for immediate affect regulation (1). Adolescents with NSSI often experience intense negative affect; the inability to tolerate this distress—compounded by high impulsivity—drives them to choose the immediate reward of relief (e.g., self-injury) over the delayed reward of long-term emotional stability (8).

A novel contribution of this study is the characterization of time perception in NSSI. Contrary to studies in depression that often report a slowing of subjective time (39), our NSSI group consistently underestimated time intervals. According to internal clock models, this pattern may reflect a slower pacemaker rate. In addition, the attentional gate model (40) suggests that reduced attention to time can also lead to underestimation. Given that adolescents with NSSI have been shown to exhibit deficits in attentional control and executive function (41, 42), it is plausible that reduced attentional resources allocated to time processing contribute to the observed underestimation. Thus, the present findings are consistent with an attentional account of time perception in NSSI. Regression analysis showed that time perception deficits predicted NSSI independently of impulsivity, suggesting distinct cognitive mechanisms. One plausible pathway involves a mismatch between expected and actual duration of negative affect. Individuals with NSSI may underestimate how long distress will last, so they would expect relief sooner than it arrives. When the expected relief does not come on time, frustration may prompt immediate action such as self-injury.

Our fNIRS results revealed that adolescents with NSSI exhibited significant hypoactivation in the L-DLPFC during the Oddball task. Early interpretations linked the L-DLPFC directly to inhibition, but influential models identify the right-lateralized fronto-basal ganglia network as critical for response inhibition (43). Inhibitory control is not an isolated mechanism but the final step in a chain of decision-making processes (44), and the DLPFC plays a well-established role in executive control (45). The observed hypoactivation may therefore reflect reduced task engagement in the NSSI group, consistent with our finding of elevated impulsivity and suggesting that these adolescents struggle to exert the cognitive control necessary to override impulsive urges. However, given that the task contrast (task blocks vs. rest) captures a broad range of cognitive processes, the specific cognitive processes reflected by this activation pattern cannot be definitively determined.

This interpretation aligns with recent evidence challenging a uniform inhibitory deficit in NSSI. Prior studies have shown that reactive inhibitory control can be enhanced in adolescents with NSSI compared to typically developing controls (41, 46), suggesting that self-harm is not driven by a failure to withhold action but rather represents a goal-directed behavior that alleviates overwhelming negative affect. Extending this perspective, our findings reveal that reduced L-DLPFC activation and reduced task accuracy during a cognitive control task co-occur in NSSI, pointing to broader difficulties in executive control.

A novel finding of this study was the hyperactivation of the Subcentral Area (SCA, Channel 22) in the NSSI group during the Oddball task. Anatomically, the SCA (Brodmann Area 43) is situated at the junction of the precentral and postcentral gyri and has known connections to sensory processing regions (47). While the functional significance of this hyperactivation in the context of a non-emotional cognitive task remains to be clarified, it may reflect altered sensory processing in adolescents with NSSI. Further research using tasks specifically designed to probe somatosensory or emotional processing is needed to understand the potential role of SCA hyperactivation in NSSI.

## 5 Limitations

It is important to acknowledge that the two-choice Oddball task employed in this study is not a pure measure of inhibitory control. While performance on this paradigm involves an inhibitory component (i.e., overriding the prepotent response to frequent stimuli), it also relies heavily on other executive processes, including attentional orienting, working memory updating, response selection, and conflict monitoring. Consequently, the reduced accuracy and altered prefrontal activation observed in the NSSI group cannot be uniquely attributed to a deficit in response inhibition but should be interpreted as reflecting broader difficulties in executive control. Future studies employing more specific paradigms, such as the Stop-Signal Task, are warranted to disentangle the various components of executive control in NSSI.

Besides, our fNIRS analysis did not employ short-channel regression to correct for superficial hemodynamic contamination. Although we used standard preprocessing procedures (HRF smoothing, wavelet-MDL detrending) to minimize physiological artifacts, future studies should consider incorporating short-channel regression to improve signal specificity.

## 6 Clinical implications

Given the associations between distorted time perception, impulsivity, and NSSI severity, cognitive training targeting temporal discrimination and estimation may help individuals better tolerate delays and support adaptive decision-making. Although L-DLPFC activation was not an independent predictor of NSSI severity, its association with time estimation errors and NSSI suggests it warrants further attention. This region may represent a potential target for neuromodulation, though causal links require confirmation.

## 7 Conclusion

Our research found that adolescents with NSSI exhibit a distinct neurocognitive phenotype characterized by high impulsivity, distorted time perception, L-DLPFC hypoactivation, and SCA hyperactivation. These findings highlight a neurocognitive pattern involving prefrontal hypoactivation and altered time perception in adolescents with NSSI, pointing to the need for further research into the underlying mechanisms.

## Data Availability

The raw data supporting the conclusions of this article will be made available by the authors, without undue reservation.
